# P-1281. Genomic and Transcriptional Adaptations in a Spontaneous Cefiderocol-Resistant Klebsiella pneumoniae KPC-Producing Mutant Isolate

**DOI:** 10.1093/ofid/ofaf695.1471

**Published:** 2026-01-11

**Authors:** Irene Luu, Vyanka Mezcord, Jenny Escalante, German Traglia, Marisel Tuttobene, Cecilia Rodriguez, Marcelo Tolmasky, Robert A Bonomo, Gauri G Rao, Fernando Pasteran, Maria Soledad Ramirez

**Affiliations:** CSUF, Fullerton, California; California State University Fullerton, Fullerton, California; California State University Fullerton, Fullerton, California; CENUR Litoral Norte, Universidad de la República, Salto, Salto, Uruguay; Instituto de Biología Molecular y Celular de Rosario (IBR), Rosario, Santa Fe, Argentina; CERELA, Tucuman, Tucuman, Argentina; CSUF, Fullerton, California; Case Western Reserve University/ Louis Stokes Cleveland VA Medical Center, Cleveland, OH; USC Alfred E. Mann School of Pharmaceiti, Rancho Palos Verdes, CA; ANLIS-Malbrán, Buenos Aires, Ciudad Autonoma de Buenos Aires, Argentina; California State University Fullerton, Fullerton, California

## Abstract

**Background:**

Carbapenem-resistant *Klebsiella pneumoniae* CRKP) represents a critical public health threat, with limited treatment options due to its resistance to last-line antibiotics. Cefiderocol (FDC), a novel siderophore cephalosporin, has shown efficacy against CRKP; however, resistance has emerged. This study characterizes a spontaneous FDC-resistant subpopulation (IHC216) derived from a KPC producing *K. pneumoniae* strain (KPNMA216). To understand this phenotype, we focused on genomic, transcriptional, and phenotypic adaptations.

**Figure 1.**
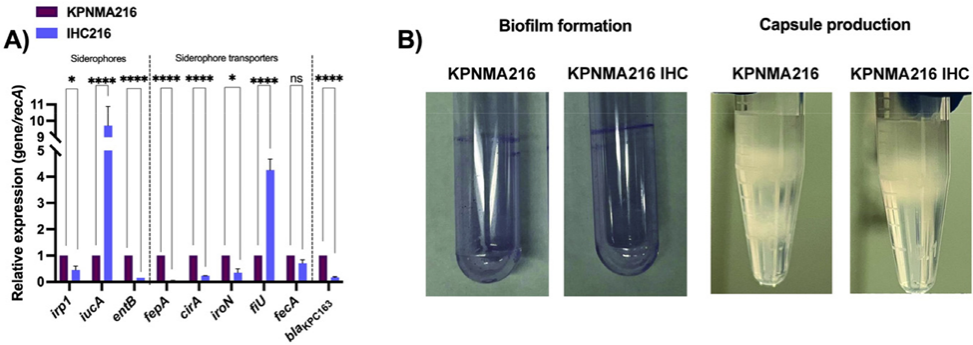
(A) Expression of genes coding for siderophores (irp1, iucA and entB) and siderophores transporters (fepA, cirA, iroN, fiU and fecA) and β-lactamase blaKPC163 in the KPNMA216 and IHC216 strains. The data shown of qRT-PCR are mean ± SD. Fold changes were calculated using ΔΔCt analysis. At least three independent biological samples were tested using four technical replicates. Statistical significance (P < 0.05) was determined by two-way ANOVA followed by Tukey's multiple comparison test using GraphPad Prism (GraphPad software, San Diego, CA, USA). Significance was indicated by: *P < 0.05, **P < 0.01, ***P < 0.001, and **** P < 0.0001. (B) Biofilm formation in tubes quantified by crystal violet and capsule density in KPC216 and IHC216 strains. three independent biological samples were tested using four technical replicates. A representative image is shown. Statistical analysis was determined by t test (p < 0.05), using GraphPad Prism (GraphPad software, San Diego, CA, USA).

**Methods:**

Whole-genome sequencing (WGS) was performed to identify genetic mutations associated with FDC resistance. Quantitative real-time PCR (qRT-PCR) was used to assess gene expression changes related to iron acquisition, antibiotic resistance, oxidative stress response, and cell wall synthesis. Antimicrobial susceptibility testing was conducted using minimum inhibitory concentration (MIC) assays. Capsule and biofilm formation were evaluated using standard biochemical assays.

**Results:**

IHC216 exhibited an increase in FDC MIC compared to the wild-type strain (from 8 to 32 ug/ml). While FDC resistance developed, meropenem MIC decreased from 32 mg/L to 0.5 mg/L, and imipenem MIC from 48 mg/L to 3 mg/L, demonstrating collateral susceptibility. WGS identified mutations in genes linked to transcriptional regulation and membrane permeability. qRT-PCR analysis revealed significant downregulation of key iron acquisition genes and upregulation of alternative iron uptake pathways (Fig.1a). *bla*_KPC-163_ expression was markedly reduced, correlating with restored susceptibility to carbapenems (Fig. 1a). Additionally, increased capsule production and biofilm formation were observed (Fig.1b).

**Conclusion:**

This study highlights the intricate genetic and transcriptional adaptations underlying FDC resistance in KPC-producing

*K. pneumoniae*. The observed collateral susceptibility to carbapenems offers potential treatment strategies that exploit this vulnerability. Understanding these resistance mechanisms is critical for optimizing therapeutic approaches against CRKP infections.

**Disclosures:**

Robert A. Bonomo, MD, Merck: Grant/Research Support|Shinogi: Grant/Research Support|VenatoRx: Grant/Research Support

